# A combination of electrochemistry and mass spectrometry to monitor the interaction of reactive species with supported lipid bilayers

**DOI:** 10.1038/s41598-020-75514-7

**Published:** 2020-10-29

**Authors:** M. Ravandeh, H. Kahlert, H. Jablonowski, J.-W. Lackmann, J. Striesow, V. Agmo Hernández, K. Wende

**Affiliations:** 1 Institute of Biochemistry, University of Greifswald, Felix-Hausdorff-Str. 4, 17489 Greifswald, Germany; 2 Leibniz-Institute for Plasma Science and Technology, ZIK Plasmatis, Felix-Hausdorff-Str. 2, 17489 Greifswald, Germany; 3 Department of Chemistry-BMC, Uppsala University, Husargatan 3, 75123 Uppsala, Sweden; 4 Department of Pharmacy, Uppsala University, Husargatan 3, 75123 Uppsala, Sweden

**Keywords:** Plasma physics, Lipid peroxides, Membrane lipids, Electrochemistry

## Abstract

Reactive oxygen and nitrogen species (RONS), e.g. generated by cold physical plasma (CPP) or photodynamic therapy, interfere with redox signaling pathways of mammalian cells, inducing downstream consequences spanning from migratory impairment to apoptotic cell death. However, the more austere impact of RONS on cancer cells remains yet to be clarified. In the present study, a combination of electrochemistry and high-resolution mass spectrometry was developed to investigate the resilience of solid-supported lipid bilayers towards plasma-derived reactive species in dependence of their composition. A 1-palmitoyl-2-oleoyl-sn-glycero-3-phosphocholine (POPC) lipid bilayer was undisturbed by 200 µM H_2_O_2_ (control) but showed full permeability after CPP treatment and space-occupying oxidation products such as PoxnoPC, PAzePC, and POPC hydroperoxide were found. Electron paramagnetic resonance spectroscopy demonstrated the presence of hydroxyl radicals and superoxide anion/hydroperoxyl radicals during the treatment. In contrast, small amounts of the intramembrane antioxidant coenzyme Q10 protected the bilayer to 50% and LysoPC was the only POPC derivative found, confirming the membrane protective effect of Q10. Such, the lipid membrane composition including the presence of antioxidants determines the impact of pro-oxidant signals. Given the differences in membrane composition of cancer and healthy cells, this supports the application of cold physical plasma for cancer treatment. In addition, the developed model using the combination of electrochemistry and mass spectrometry could be a promising method to study the effect of reactive species or mixes thereof generated by chemical or physical sources.

## Introduction

Reactive oxygen and nitrogen species, able to interfere with cell and tissue-wide redox signalling pathways, gain increasing attention as tools to control and modulate cellular processes^1,2^. Examples are the long established photodynamic therapy that is used to remediate (pre-) cancerous lesions^3^, and diverse drugs, e.g. doxorubicin^4^, platinum complexes^5^, or celecoxib^6^ that are used or under test as anti-cancer drugs. In contrast, cold physical plasma^7,8^ (CPP) is an emerging technology that is being used for biomedical purposes like wound healing^9–13^ (clinical use), treatment of skin related disorders including precancerous lessions^14,15^ (case studies), and cancer^16–19^ (case studies, models). Cold plasmas generate a variety of reactive oxygen (ROS, e.g., ^·^OH, ^1^O_2,_ H_2_O_2_, O_2_^·−^, ^·^O, O_3_) and nitrogen species (RNS, e.g., ^·^NO, ^·^NO_2_, ONOO^-^) that are partly deposited in the treated matter, such as a liquid, or the tissue. Due to current understanding, it provides an (pro-) oxidative environment surrounding the biological target mediating effects on the signal protein expression, cell cycle progression, cell metabolism including mitochondria, and immune footprint of cells^17,20,21^. Various studies have indicated that CPPs modulate intracellular reactive oxygen species (ROS) levels, thus inducing oxidative damage in mammalian cells, which can lead to cell death, i.e., apoptosis^22,23^. Experimental studies emphasize a more severe impact of plasma-derived ROS/RNS on cancer compared with normal cells^24,25^. It is known that healthy and cancer cells differ in several aspects, e.g. levels of intracellular antioxidants, metabolic strategies, and the plasma membrane composition^26^, suggesting a more effective protection from the potentially deleterious impact of ROS/RNS in normal cells^27,28^.

As the primary interaction site of short-lived reactive species derived from CPP the cytoplasmic membrane is discussed^29–31^. This highly selective barrier is formed at the core from complex lipids whose composition varies significantly with cell type and physiologic condition and it represents a major hub of cellular signalling cascades responding to external (oxidative) stimuli that can be modulated by cellular antioxidants. One of the main intramembrane antioxidants is coenzyme Q, an intriguing family of lipophilic molecules present in the intra-membranes and cytoplasm of most eukaryotic cells and in the membranes of gram-negative bacteria^32–34^. The predominant variant in human cells is coenzyme Q10 (Q10). Q10 is associated with several aspects of the cellular metabolism, in particular the mitochondrial respiration chain, in which it plays a significant role in the electron and proton transport across lipid membranes. Moreover, it acts as a powerful antioxidant protecting the membrane lipids from peroxidation^35–37^. Recent finding suggest that the Q10 also enhances the mechanical stability of the cell membrane^32,38–40^. Lower concentrations of coenzyme Q10 have been demonstrated in cancer cell lines and in sera of cancer patients^41,42^.

Lipid vesicles^43–45^, supported lipid bilayers (SLB)^46–49^, and freestanding lipid bilayers^50^ can provide a valuable in vitro-platform for the investigation of a variety of biological questions associated with cell membrane function, structure, and composition^51,52^. Of those, SLB as a lipid bilayer film on solid supports can mimic the key architectural features of cellular membranes. Besides the broad range of applications, it has advantages over other techniques^53–55^. One of the most powerful benefits of SLBs is that they are compatible with a variety of surface-sensitive techniques such as electrochemical methods, atomic force microscopy, and quartz crystal microbalance^56–58^. Several methods have been developed for the fabrication of SLB, e.g. Langmuir–Blodgett and Langmuir–Schaefer, vesicle fusion, and solvent assisted methods^59^. Especially the vesicle fusion method is widely used for its versatility and speed. It involves the adsorption and rupture of small unilamellar vesicles (smaller than 100 nm diameter) on a ultra-flat solid surfaces^60^.

There are various methods for evaluation the effect of reactive species on cellular biomolecules, such as spectrophotometry, mass spectrometry, chemiluminescence, and electrochemistry^61–63^. Among these, the application of electrochemistry is one of the best, because the interaction between reactive species and biomolecules based on electron transfer reactions can be easily monitored^64,65^. On the other hand, mass spectrometry method provides specific information on the effect of reactive species on the composition of biomolecules^30^.

**Figure 1:**
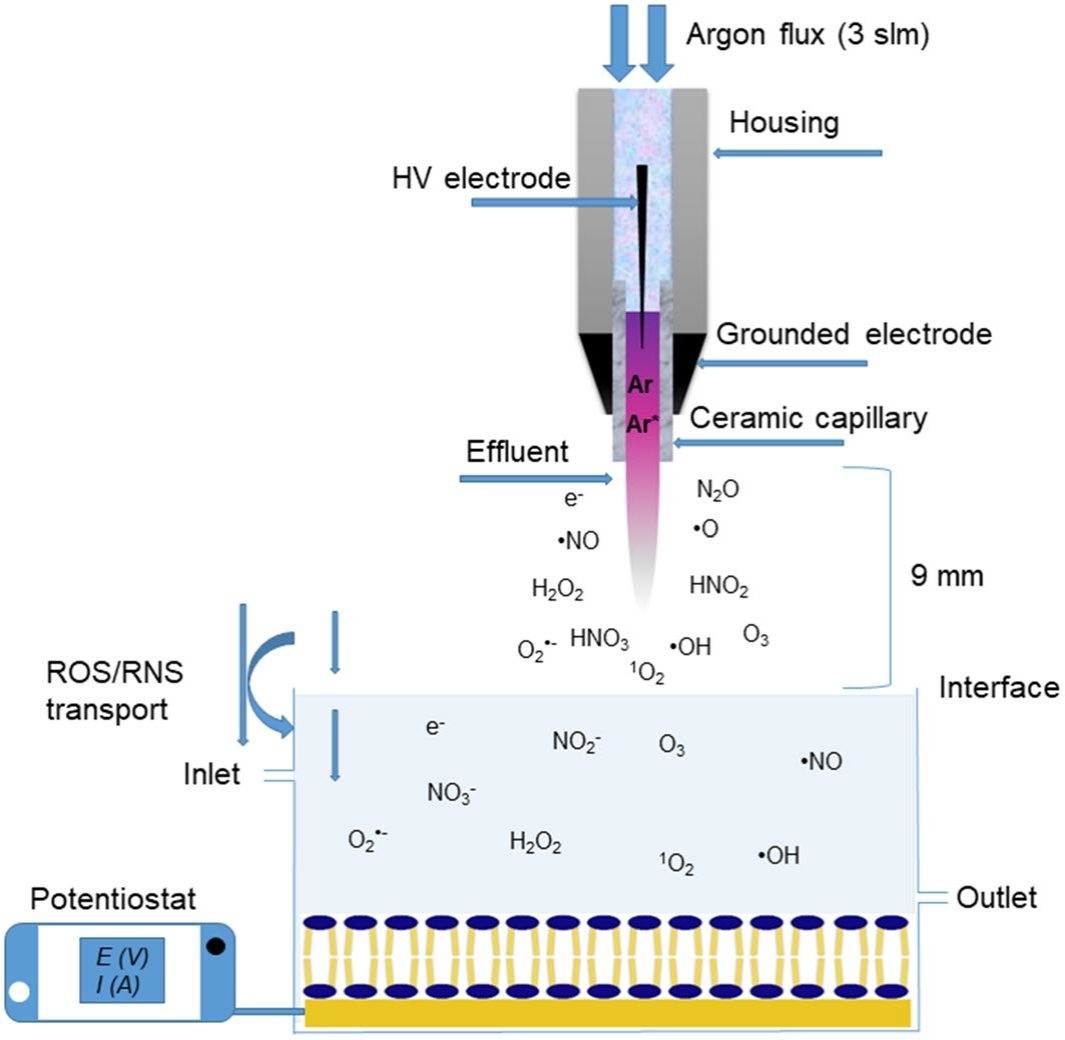
Schematic of electrochemical flow cell and treatment of a lipid bilayer by reactive species generated by cold physical plasma.

In the current study, a combination of electrochemical measurements and mass spectrometry was developed and applied to infer on changes in both functionality and chemical composition of the SLB resulting from CPP and adjacent sources derived reactive species attack. Special attention was given to the fact whether plasma-derived species are active beyond the gas–liquid interface. To this end, a gold supported lipid bilayer was prepared by the potential cycle assisted vesicle fusion method^66^. Two different model membranes were studied, POPC and POPC:Q10 mixed lipid bilayer, to decipher the role of intramembrane antioxidants during impact of reactive species from external sources (Fig. 1).

## Results and discussion

### Fabrication of solid supported lipid bilayer

**Figure 2:**
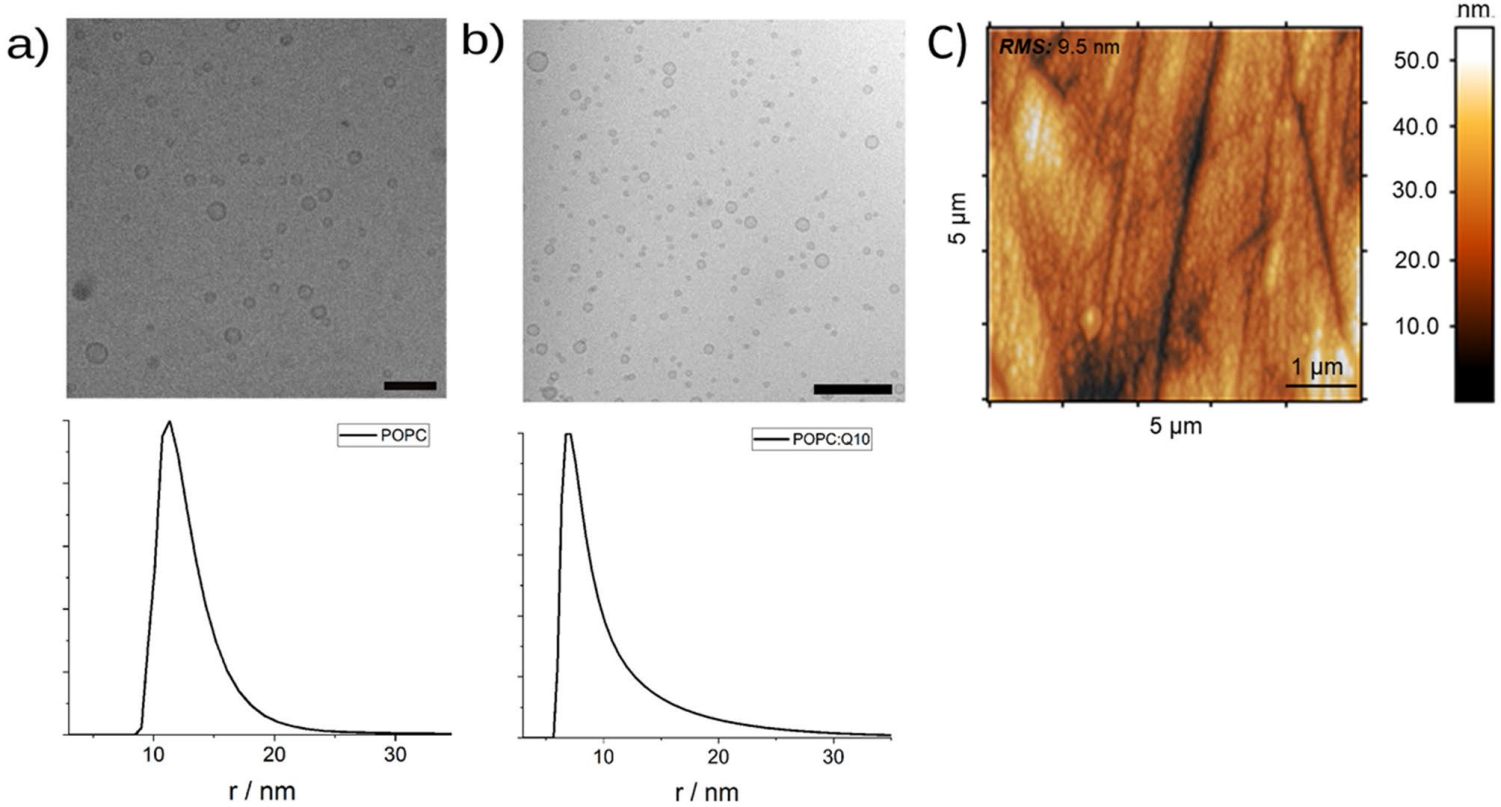
Cryo-TEM (bar: 100 nm) and DLS of SUVs of POPC (**a**) and POPC:Q10 (**b**), and surface topography of Au electrodes after mechanical and electrochemical cleaning procedures suitable for vesicle fusion method (**c**).

Liposomes as a starting material were prepared by sonication, and characterization by DLS and Cryo-TEM confirmed that SUVs with an average radius of 13 ± 4 nm for pure POPC (Fig. 2a) and 10 ± 5 nm for the POPC:Q10 (Fig. 2b) have formed. The necessary ultra-flat gold surfaces were obtained by a mix of mechanical and electrochemical treatments; the achieved average roughness of gold electrode is 9.5 ± 1.3 nm as characterized by AFM (Fig. 2c).

**Figure 3:**
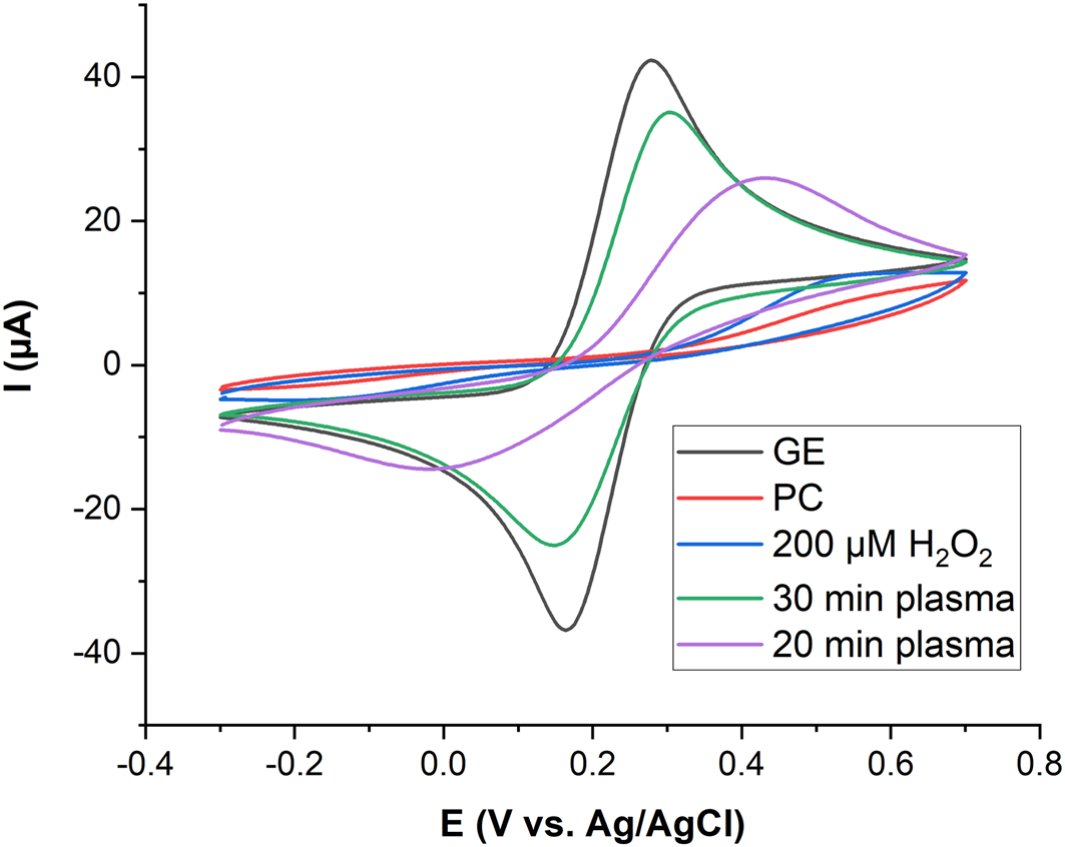
Cyclic voltammograms of 10 mM K_4_[Fe(CN)_6_] in 50 mM phosphate buffer (scan rate 50 mV s^−1^) for bare gold electrode (GE), after formation of POPC lipid bilayer (PC), after direct plasma treatment of the SLB (20 min/30 min plasma), or with 200 µM hydrogen peroxide (200 µM).

Supported lipid bilayer was prepared by potential assisted vesicle fusion method. It has been reported that the structure of a lipid bilayer on a gold support changes according to the applied potential^67^. Slow scan rates allow the system to reach the equilibrium structure of the immobilized lipid layers at each potential. Repetitive prolonged scans result in the rupturing of adsorbed liposomes and the formation of a lipid bilayer. For Q10-containing bilayers, the slow scan rates are necessary to assure the complete reduction/oxidation of Q10 during the voltammetric cycle^68^. The formation of lipid bilayer on gold electrode was characterized by cyclic and differential pulse voltammetry of the ferrocyanide/ferricyanide redox system. For bare gold electrode, voltammetric methods show the oxidation and reduction peaks of the redox system but after formation of the lipid bilayer on gold surface, the peaks vanished, indicating the full coverage of the electrode by a lipid bilayer (Fig. 3).

### Coenzyme Q10 incorporated in supported lipid bilayer

**Figure 4:**
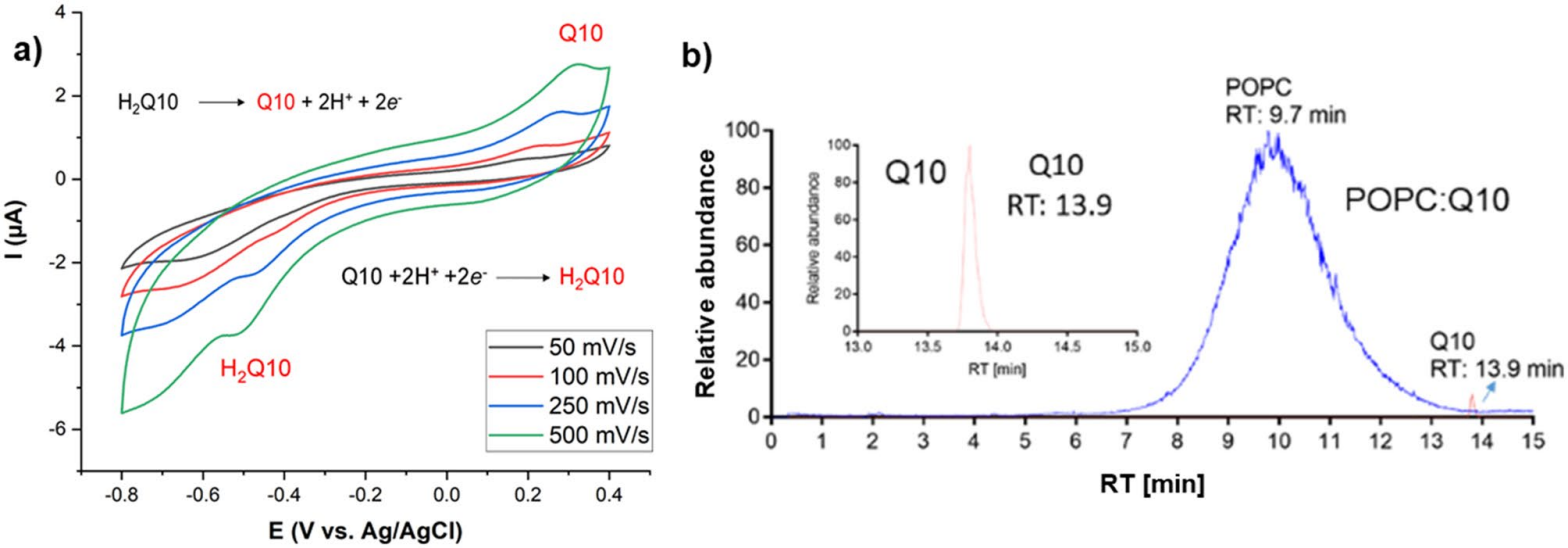
Cyclic voltammograms of Q10 incorporated into lipid bilayer at different scan rates in presence of 10 mM HEPES buffer (pH 7.4) as the electrolyte (**a**) and LC/MS extracted ion chromatogram (XIC) of authentic coenzyme Q10 and POPC:Q10 (25:1 mol ratio) lipid bilayer extract (**b**) proofing the successful incorporation. Both precursor ions shown were detected in positive ionization mode in their protonated form [M + H]^+^.

The presence of Q10 in lipid bilayer was monitored by cyclic voltammetry and LC/MS methods. The cyclic voltammograms of Q10 incorporated in the lipid bilayer at different scan rates in presence of 10 mM HEPES buffer (pH 7.4) as the electrolyte showed clear oxidation (*E*_p,a_ =  + 0.25 V) and reduction (*E*_p,c_ =  − 0.5 V) peaks corresponding to Q10 inside the lipid bilayer (Fig. 4a). The obtained voltammograms show the characteristic features (wide peak separation and broad peaks) reported by (Jeuken et al.)^69^ for Q10 incorporated in tethered bilayers and by (Martensson and Agmo Hernandez)^66^ for a POPC:Q10 SLB supported on gold under similar conditions to the ones explored in this report. We can conclude that Q10 was successfully incorporated into the lipid bilayer. Moreover, LC/MS experiments confirm the incorporation of Q10 by showing the extracted ion chromatogram (XIC) of authentic coenzyme Q10 and POPC:Q10 lipid bilayer extract in positive mode with identical retention times (RT: 13.9 min) and m/z 863.69 for [Q10 + H]^+^ (Fig. 4b) and MS1/MS2 traces like authentic Q10 showing a diagnostic fragment ion at m/z 197 (Fig. S1), corroborating the results obtained by electrochemical techniques.

### Electrochemical analysis of POPC and POPC:Q10 SLBs after plasma treatment

To confirm whether the reactive species generated by CPP exhibit the ability to induce oxidative lesions in the lipid bilayer on the gold electrode, cyclic voltammograms were recorded before and after plasma treatment. According to results (Fig. 3 and Table 1), after 20 min plasma treatment, there is an increase in the oxidation and reduction peaks of the ferrocyanide/ferricyanide redox system, suggesting the lipid degradation due to oxidative damage caused by CPP derived reactive species. However, the wide peak separation (0.365 ± 0.027 V) indicated the redox system can diffuse into the lipid bilayer but the ions are not able to travel through lipid bilayer to reach the gold electrode. In contrast, at 30 min, there is a fast electron transfer between the electrode and the redox system, and the peak separation decreased to a value very closed to that of the redox system at bare gold electrode (0.148 ± 0.005 V and 0.142 ± 0.016 V) indicating a rupture of the lipid bilayer or a significant pore formation^63^. One can assume that the CPP-derived reactive species induces oxidative changes in the POPC molecule and with increasing time, a change in the SLB structure occurs leading to an increased permeability. The observed two-phase behaviour—initially a slow increase of the permeability followed by a sharp increase of electron transfer could be explained by a slow loss of the ordered POPC lipid bilayer, rendering parts of the SLB more penetrable, followed by a localized or generalized collapse of the bilayer structure.

**Table 1:** Electrochemical parameters of 10 mM K_4_[Fe(CN)_6_] in 50 mM phosphate buffer at bare gold electrode, and for lipid bilayer modified gold electrode after 20 and 30 min plasma treatment.

	I_ox_ (µA) ± SD	I_red_ (µA) ± SD	ΔE (mV) ± SD
Bare electrode	39.1 ± 2.7	39.6 ± 2.0	142 ± 16
SLB 30 min CPP	28.9 ± 1.0	27.1 ± 1.0	148 ± 5
SLB 20 min CPP	20.6 ± 2.6	12.2 ± 0.5	365 ± 27

The introduction of polar groups into the side chain of the oleic acid residues by reactive species would explain this observation, assuming that ultimately either the number of polar side chains is too large to maintain the overall membrane structure or additional head group modifications lead to molecule cleavage and structure loss^30,65,70,71^. Interestingly, the long-lived ROS hydrogen peroxide in a concentration deposited during 30 min of plasma treatment (100 µM) could not oxidize the POPC bilayer in the same extent (Fig. 3), indicating that the short lived species produced by the CPP play the major role^30^.

In normal conditions, cells are provisioned with numerous sensors and defence systems to measure and prevent undesired effect of reactive species regardless of their origin^30,72^. In the cytoplasmic and mitochondria membranes, coenzyme Q10 (in its reduced form ubiquinol) is an important intramembrane antioxidant and an established factor in membrane protection^73^. However, it has been suggested that the oxidized form of lipid soluble antioxidants provides protection against pro-oxidants via structural interactions with the lipids, decreasing the solute and water permeability of lipid membranes, while increasing the membrane rigidity and resistance to rupture^32,38,40^. The increase in mechanical stability of the membrane results in a decreased penetration of reactive oxygen species (ROS) into the membrane, decreasing the oxidation of lipid tails in bilayers supplemented with Q10 even if the molecule is found in its oxidized state (Fig. 4).

**Figure 5:**
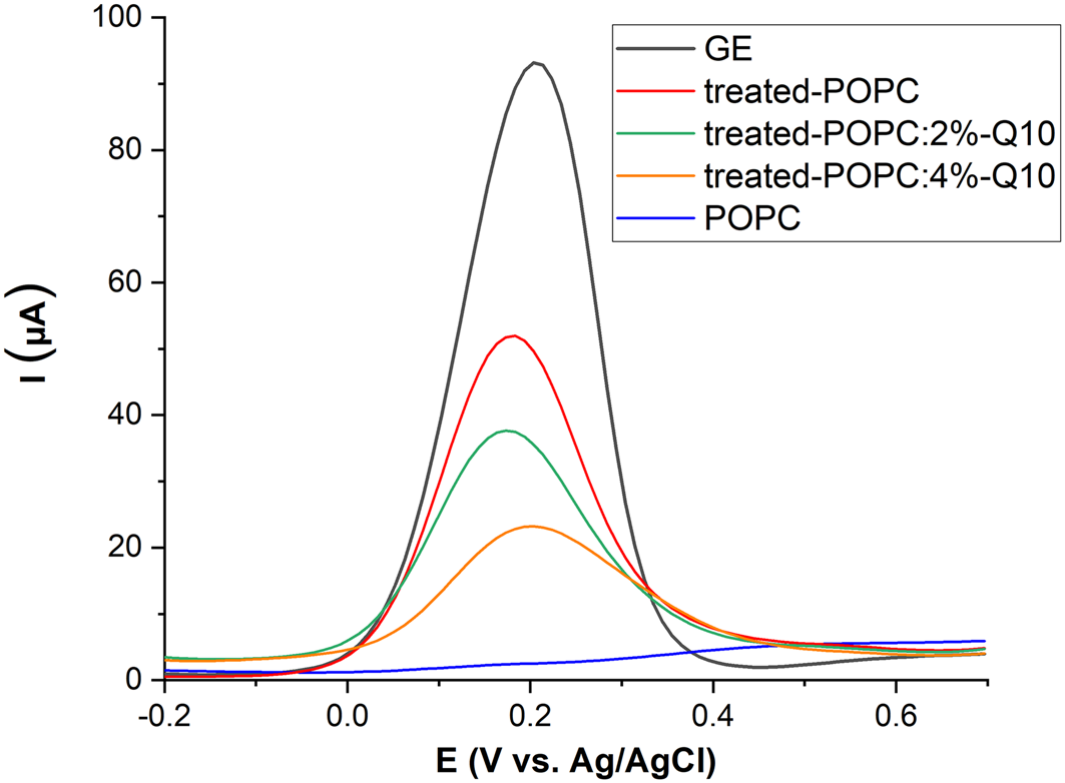
Differential pulse voltammograms of 10 mM K_4_[Fe(CN)_6_ in 50 mM phosphate buffer (scan rate = 50 mV s^−1)^, for clean gold electrode (GE), after POPC SLB formation (POPC), and after 30 min plasma treatment of lipid bilayer in the absence (treated POPC) or presence of 2% and 4% Q10 (treated POPC:2/4%-Q10). The protective effect (up to 53%) of Q10 is obvious (see text).

In contrast, an increased head group oxidation might be expected but was not observed. Indeed, during the plasma treatment of POPC:Q10, a lower increase in the anodic current of the oxidation of [Fe(CN)_6_]^4−^ was recorded in comparison with the current observed in the absence of Q10, and a protective effect of 52.6 ± 2.4% and 19.3 ± 3.8%was observed for 4% and 2% Q10, respectively (Fig. 5). The percentage of protection effects were calculated by using the following equation:

$$ \% {\text{ Protection effect}} = \left[ {\left( {I_{{1}} {-}I_{{2}} } \right)/I_{{1}} } \right] \times {1}00 $$

where *I*_1_ and *I*_2_ are the electrochemical currents obtained after plasma treatment of lipid bilayer without and with Q10, respectively. These observations confirm the ability of the Q10 in protection of lipid bilayer during plasma treatment.

### Covalent changes to POPC due to CPP-derived species

**Figure 6:**
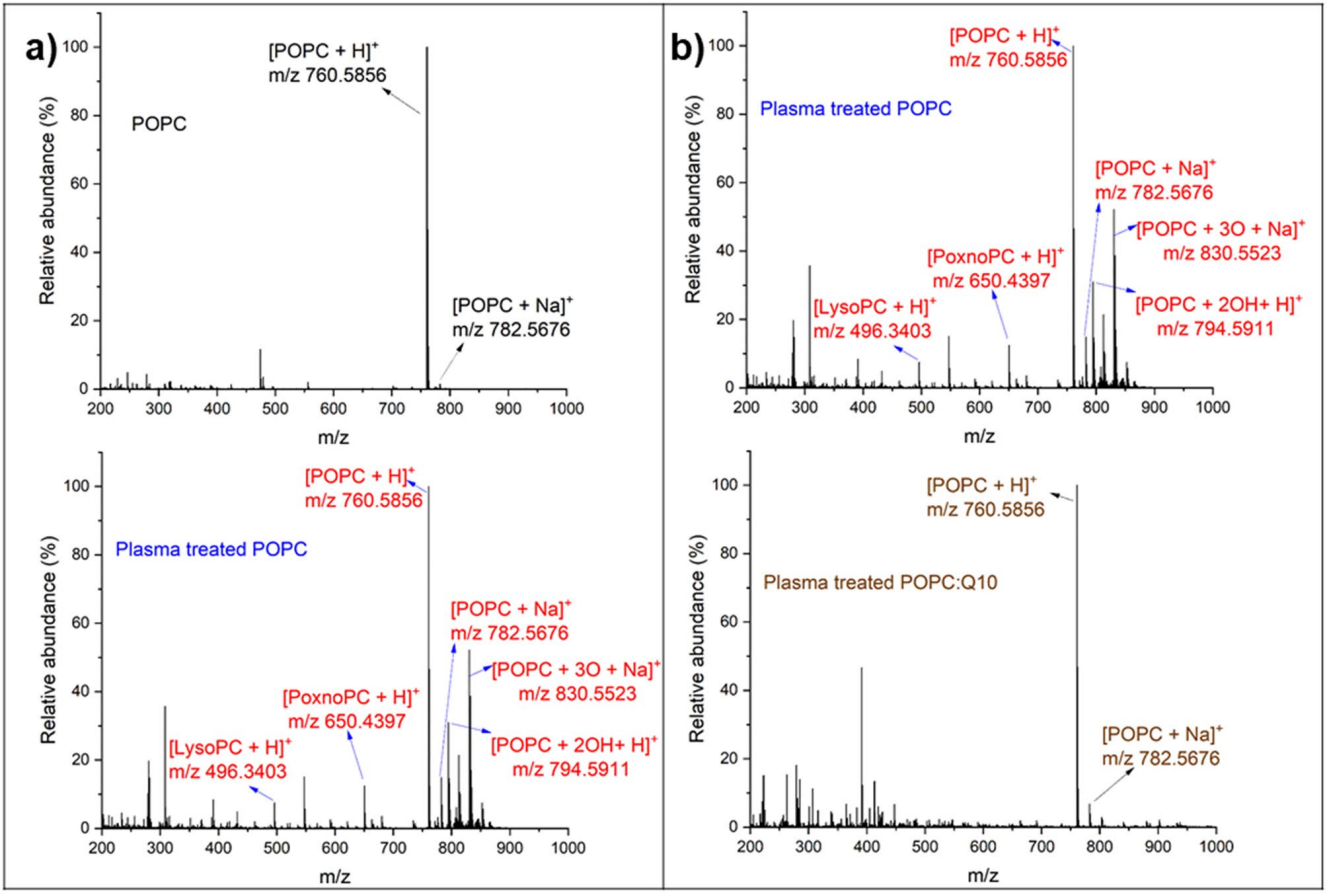
Mass spectrometry of POPC extracts, before (POPC) and after 30 min plasma treatment (plasma-treated POPC) (**a**) reveal presence of POPC oxidation products ([LysoPC + H]^+^ (m/z 496.3403) (C_24_H_51_NO_7_P), [PoxnoPC + H]^+^ (m/z 650.4397) (C_33_H_65_NO_9_P), [POPC + 2OH + H]^+^ (m/z 794.5911) (C_42_H_85_NO_10_P), [POPC + 3O + Na]^+^ (m/z 830.5523) (C_42_H_82_NNaO_11_P)), Mass spectrometry of POPC and POPC:Q10 (25:1 mol ratio) after 30 min plasma treatments (**b**).

**Figure 7:**
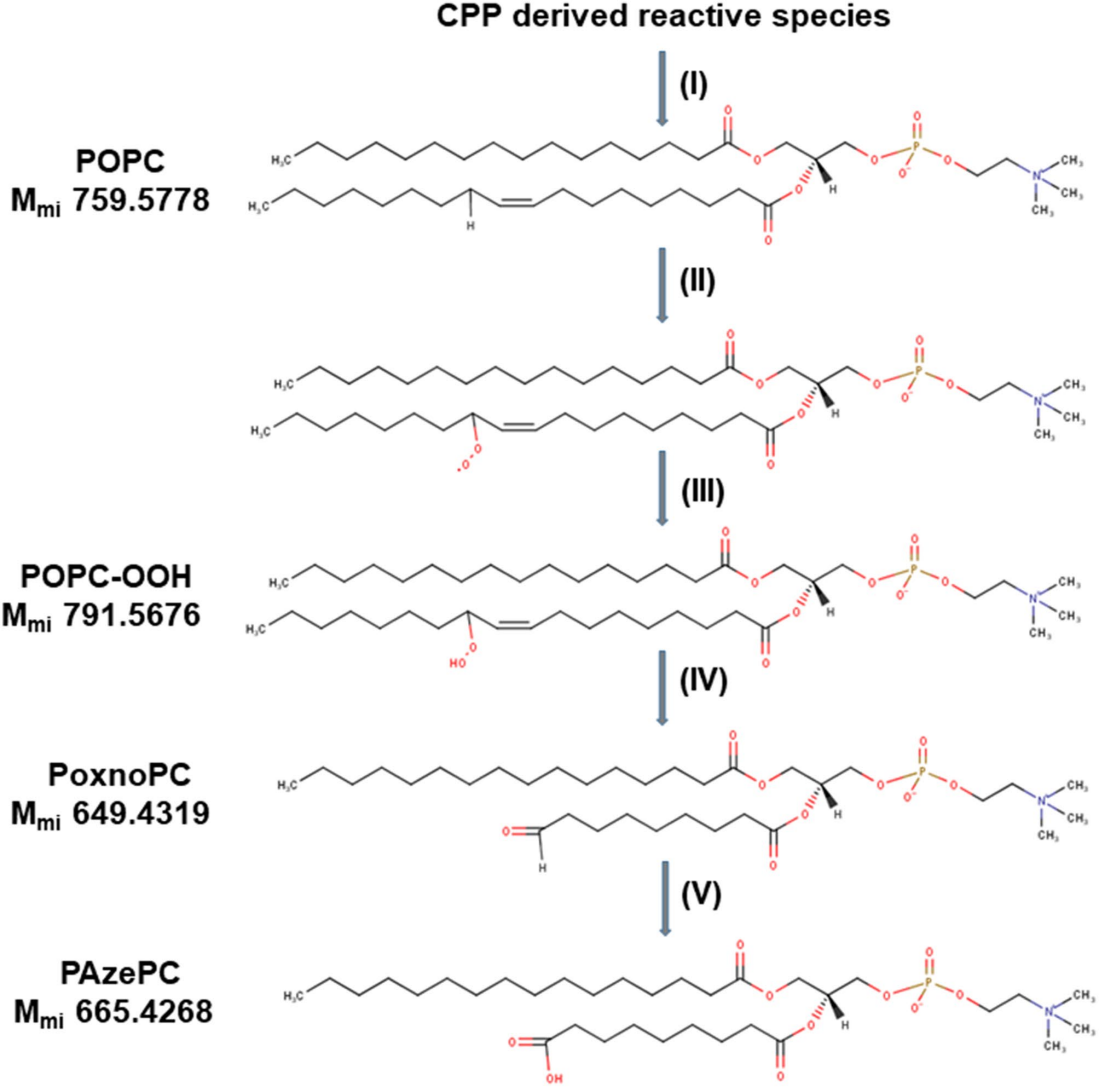
The postulated mechanism of POPC lipid bilayer oxidation. (I) The allylic hydrogen is abstracted by CPP derived reactive species and (II) with the addition of molecular oxygen, the hydroperoxyl radical is added in its place. (III) The hydroperoxyl radical can initiate the oxidation of neighboring lipid molecules to result in a hydroperoxy (OOH) group adjacent to the double bond. (IV) Oxidation of the OOH group results in lipid tail scission into a shortened acyl chain capped with the aldehyde (CHO) group. (V) Further oxidation of the shortened lipid molecule results in its replacement CHO with COOH. M_mi_ = monoisotopic mass.

The chemical integrity of POPC was analysed using high-resolution mass spectrometry. Comparing the mass spectra obtained by the direct infusion of SLB MTBE extracts before (control) and after plasma treatment, additional peaks were found (Fig. 6a). In control, the positive mass spectrum shows the molecular ion of the native POPC as [POPC + H]^+^ (m/z 760.6) and the common adduct [POPC + Na]^+^ (m/z 782.6). The presence of both ions was taken into account for the interpretation of the mass spectrum obtained after plasma treatment. After plasma treatment, additional peaks were observed indicating covalent modifications of POPC. Among these, the peaks at m/z, 496.3, 650.4, 666.4, 776.6, 794.5, and 830.5 in positive mode were of significance. Acquired fragment spectra identified the presence of the phosphocholine head group (m/z 184.07) for all peaks of interest, confirming them as oxidized POPC products (Fig. S2 (a–f)). Accordingly, the peaks at m/z 794.5, 650.4, 496.3, and 666.4 were attributed to two hydroxyl groups^74^, aldehyde, and carboxyl moieties, thereby indicating the formation of a dihydroxylated PSPC (POPC + 2OH),1-palmitoyl-2-(9′-oxo-nonanoyl)-sn-glycero-3-phosphocholine (PoxnoPC), 1-palmitoyl-2-hydroxy-sn-glycero-3-phosphocholine (LysoPC) and 1-palmitoyl-2-azelaoyl-sn-glycero-3-phosphocholine (PAzePC), respectively. In contrast to expectations from literature, no hydroperoxide at m/z 792.5749 [POPC + 2O + H]^+^ was observed. Instead, a peak at m/z 794.5911 was detected indicating the presence of two additional hydrogen atoms in the molecule. Negative mode tandem mass spectra indicated the presence of two hydroxyl groups in the side chain, alongside with the loss of the double bond (m/z 315 in neg. mode, see proposed structure in Figure S2/E). Assumingly an epoxy lipid is formed by the attack of a peroxide and is subsequently hydrolysed by a nucleophilic attack to a ring carbon. The nature of the attacking ROS is unknown; the hydrogen peroxide produced by the plasma can be excluded since control experiments using H_2_O_2_ did not yield this product. Atomic oxygen might be a candidate species, however its life time liquids is an issue, significant migration of atomic O was observed by Omlid et al. quite recently^75^. The presence of a double bond at C-9 of POPC leads to the presence of two allylic hydrogen atoms at C-8 and C-11 that are primarily attacked by the CPP-derived reactive species (site specific “ene” chemistry)^76^. The abstraction of an allylic hydrogen atom allows the addition of CPP derived reactive species, e.g. singlet oxygen (O_2_^1^Δ_g_), atomic oxygen (O), OH radicals (^·^OH), or nitric dioxide (^·^NO_2_) released from peroxynitrous acid, yielding the respective peroxyls or alkoxyls. Subsequent rearrangements (Hock cleavage, homolytic β-scission, etc.) lead to stable products such as aldehydes (PoxnoPC), carboxylic acids (PAzePC) (Fig. 7).

**Figure 8:**
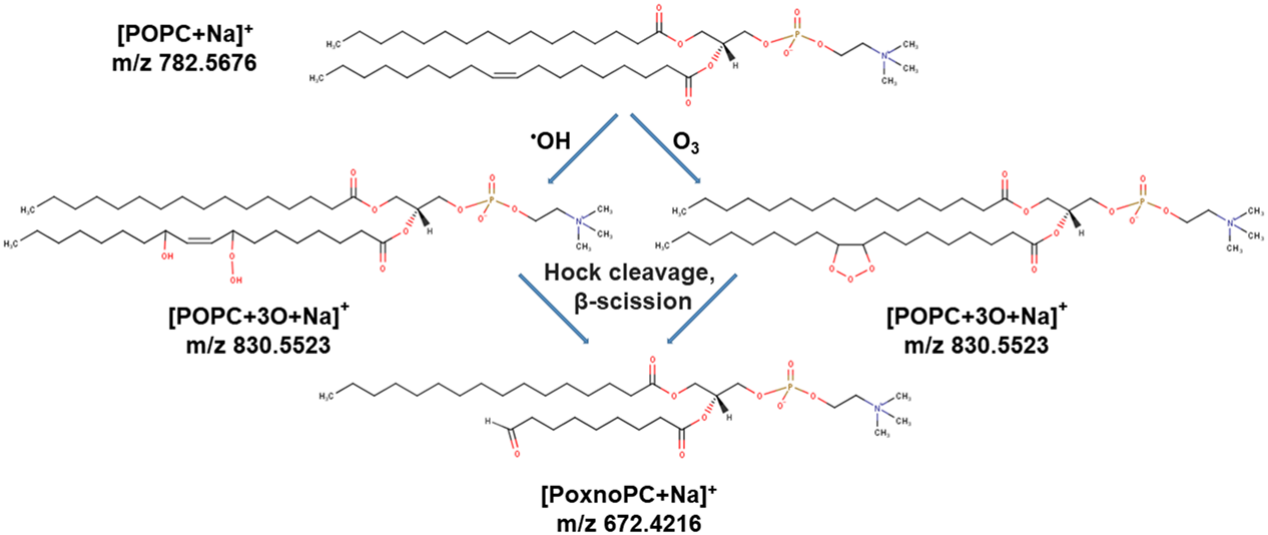
Suggested products of peak at m/z = 830.55 with addition of three oxygen atoms in lipid structure after plasma treatment and subsequent rearrangements (Hock cleavage, homolytic β-scission, etc.) lead to stable products such as aldehydes (PoxnoPC)^76,78–80^.

These identified products are in agreement with the results of NMR spectrometry based on the effect of singlet oxygen on giant unilamellar vesicles^77^ and MS data obtained by the treatment of DOPC liposomes with an argon plasma jet^30^. Of interest is the role of hydroxyl radicals: after H abstraction, addition of a second ^·^OH radical would yield a relatively stable hydroxyl group—but only traces have been observed. In addition, the peak at m/z 830.5 indicated putative insertion of three oxygen atoms. This peak was reported by Reis et al.^76^, based on the oxidation of POPC lipids by hydroxyl radicals during Fenton reaction and involving a Hock rearrangement. Moreover, Ellis et al.^78^ observed similar peaks after ozonolysis of unsaturated lipids based on Criegee mechanism of alkene ozonolysis (Fig. 8). In addition, negative mode tandem mass spectrums of all POPC oxidation products were obtained and studied ((Fig. S3 (a–f)).The peak at m/z 255.2324 belongs to palmitic acid fragment for LysoPC and other oxidation products in negative mode. Moreover, the peaks at m/z 171.1020 and 187.0970 indicated the formation of aldehyde and carboxyl moieties in oleic acid fragment. The peaks at m/z 297.2430, 315.2541, and 329.2333 were attributed to the addition of one hydroxyl, two hydroxyl, and three oxygen to oleic acid fragment of POPC. The negative mode results confirmed the presence of the proposed oxidation products of POPC based on the oxidation on alkyl chain.

These results proposed the effect of different reactive species generated by CPP such as superoxide anion radicals, hydroxyl radicals and theoretically ozone, which we can exclude due to the experimental conditions, on the lipid bilayer. Hydrogen peroxide (200 µM) was ineffective in oxidizing the POPC. When 4% Q10 were incorporated into the POPC lipid bilayer before the plasma treatment, oxidative damage to the POPC molecule is drastically reduced. No significant peaks of oxidation products of POPC were observed, only the hydrolysis product LysoPC was found in this condition (Fig. 6b and Table 2). That confirms the conclusions from the electrochemical experiments, further substantiating the membrane protection provided by Q10 against plasma-derived reactive oxygen and nitrogen species.

**Table 2:** POPC oxidation products for POPC and POPC:Q10 after plasma treatments, observed by high resolution mass spectrometry.

POPC oxidation product	m/z (positive mode)	Treated POPC	Treated POPC:Q10
[LysoPC + H]^+^	496.3403	+	+
[PoxnoPC + H]^+^	650.4397	+	−
[PAzePC + H]^+^	666.4346	+	−
[POPC + OH + H]^+^	776.5805	+	−
[POPC + 2OH + H]^+^	794.5911	+	−
[POPC + 3O + Na]^+^	830.5523	+	−

### CPP-derived reactive oxygen species in the model system

**Figure 9:**
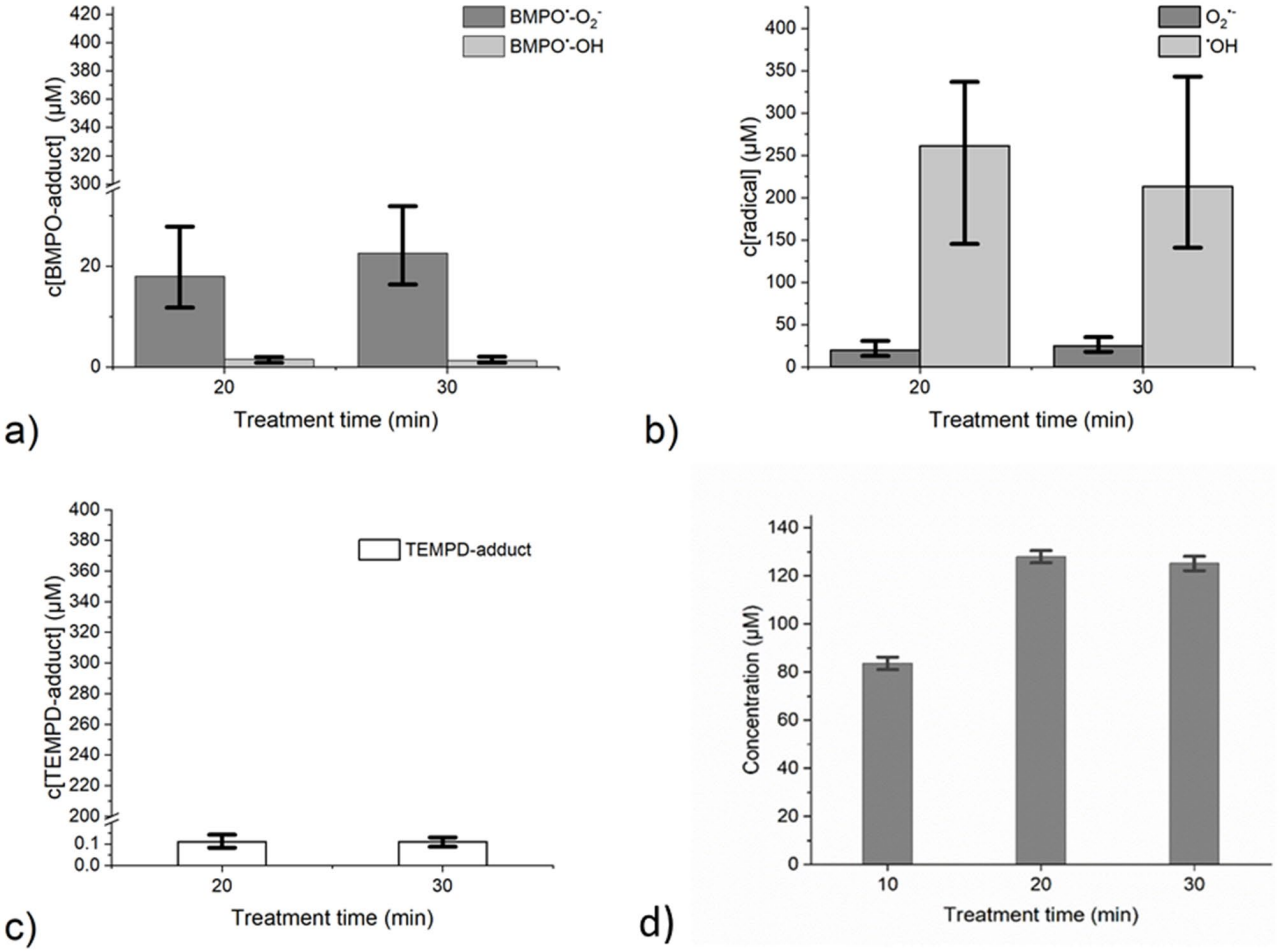
Absolute concentration of BMPO-adducts BMPO-O_2_H/BMPO-O_2_^−^ (dark gray bars) and BMPO-OH (light gray bars) as measured by EPR (**a**), calculated concentrations using a reaction probability factor superoxide anion/hydroperoxyl radicals (dark gray bars) and hydroxyl radicals (light gray bars) (**b**), absolute concentration of TEMPD-adducts of either atomic oxygen, ozone or singlet oxygen (white bars) measured by EPR (**c**), absolute concentration of hydrogen peroxide in the model system (Fox assay) (**d**), kINPen IND, 3 slm argon without shielding gas, 9 mm distance to the target, 3 experiments, error bars represent the range.

The proposed model system—a supported lipid bilayer in an aqueous system was chosen to segregate the gas–liquid interface and the bulk of the liquid. Due to the gaseous nature of the plasma, high numbers of different species are hitting the interface region, with some species, such as hydrogen peroxide, being readily absorbed in the bulk. Whether or not also short-lived species can penetrate into the liquid remains to be clarified. Candidates would be OH radicals that can extent into the liquid bulk via bond flipping mechanism^81^, or atomic oxygen that reportedly can pass a semipermeable membrane cage and react with a bait molecule outside^75^. To elucidate the potential candidate species responsible for the observed modification of POPC combined with the loss of membrane integrity, spin trap enhanced EPR spectroscopy was deployed. By using the spin trap BMPO, the accumulated presence of ^·^OH, superoxide anion (O_2_^·−^)/hydroperoxyl radicals (HO_2_^·^) was measured while the spin probe TEMPD-HCl allowed assessment of atomic oxygen, molecular oxygen in the singlet state, and ozone (Fig. 9a–c).

Taking the trapping efficacy of BMPO for the different radicals into account (^·^OH radicals 0.6% (Tong et al.)^82^, O_2_^·−^/HO_2_^·^ radicals 90% (Rosen et al.)^83^), a dominance of hydroxyl radicals was observed over superoxide anion/hydroperoxyl radicals during the plasma treatment. For a number of reasons, including transport rate limitations at the gas–liquid interface and local non-equilibrium conditions which are not reflected in the literature values this approach offers a rough estimation only, suggesting a ^·^OH radical deposition rate between 7.2 × 10^12^ s^−1^ (final 1.5 µM in experimental conditions) and 1.2 × 10^15^ s^−1^ (final 250 µM in experimental conditions), respectively. While the first value (neglecting the reaction probability factor) is low compared to measured gas phase species densities, the second value may overestimate the presence in liquids. However, the cumulative hydrogen peroxide concentration (≈ 120 µM) indicated a similar order of magnitude and could reflect the recombination of each two ^·^OH radicals to form H_2_O_2_. No significant difference was observed for 20 or 30 min of treatment, supposedly due to saturation processes and/or decay of the spin trap (Fig. 9b). The observed ^·^OH radicals are formed in the gas and the liquid phase from water molecules, cleaved by collision with higher energy species and (V)UV photons, respectively. The remaining ^·^H atoms contribute to the formation of HO_2_^·^ radicals via the reaction with molecular oxygen, yielding to O_2_^·-^ radicals under the slightly acidic experimental conditions (pH 5–6). An accumulated concentration of ≈ 25 µM was observed, translating into a deposition rate of 1.2 × 10^14^ s^−1^, fitting published gas phase densities of O_2_^·−^/HO_2_^·^^84,85^. Considering the pKa of 4.88 for HO_2_^·^, 60 to 90% will be dissociated under experimental conditions. A contribution of hydrogen peroxide as a precursor for HO_2_^·^ (via ^·^H abstraction by ^·^OH or ^·^O) cannot be excluded but the concentration of H_2_O_2_ ranges factor 10 above ^·^O_2_^-^/HO_2_^·^ radicals suggesting that its cleavage is not a major route. The number of atomic oxygen atoms reaching the interface/liquid bulk is limited in kINPen discharges (see below). Furthermore, water molecules (c = 55 mol L^−1^) have a 460,000 fold superiority over hydrogen peroxide (≈ 120 µM) reducing the chance of collision between H_2_O_2_ and short-lived radicals. Concentrations at the gas–liquid interface may be higher than in the equilibrium state of the bulk liquid, increasing the interaction potential. The cumulative TEMPD-adduct concentration confirmed a lack of treatment time dependency. To the best of the author’s knowledge, no data on the trapping efficiency of TEMPD (2,2,6,6-tetramethylpiperidine) with its targets are available and only the adduct concentration could be given (Fig. 9c). The low concentrations observed (around 0.1 µM, or ≈ 5 × 10^11^ species per second), may reflect a fast decay of the probe by hydroxyl radicals as assumed for TEMP (2,2,6,6-tetramethylpiperidine) by Privat-Maldonado et al.^86^, or a limited production of atomic or singlet oxygen as well as ozone under the treatment conditions used. The latter is in good agreement with work published previously, where relevant TEMPD adducts were found for oxygen enriched feed gas only, modulated by the distance and the presence of a nitrogen shielding gas^87^.

Ozone formation and subsequent deposition is especially favoured in long distances between plasma jet and target, for instance above 100 mm, precluding its relevance in the current conditions (< 10 mm distance) that rather favour TEMPD adduct of atomic or singlet oxygen. In a previous study, Jablonowskí et al.^87^ found that for the kINPen atomic oxygen may reach the liquid surface only if no molecular oxygen in present in the surrounding of the plasma plume, conditions that were not applied here. Nevertheless, using adapted conditions for the kINPen or plasma devices generating sufficient amount of atomic oxygen also in open air (e.g. COST-jet) would allow the interaction of atomic oxygen with targets in the bulk liquid as was shown by Benedikt et al.^88^ or Wende et al.^89^. Another plasma source also operated with helium as feed gas, similar to the COST-jet, operated in controlled environment produced mainly atomic oxygen under the investigated conditions reported by Elg et al.^90^. In the mentioned publications evidence was provided that atomic oxygen, although present in low concentration in the experimental conditions may contribute to the oxidation of the lipids, assumingly via the formation of ^·^OH and HO_2_^·^ radicals at or close to the gas–liquid interface. This notion is backed by the observation that atomic oxygen can migrate in water and leave a molecular cage to react with outside molecules^75,91^.

Given that O_3_ should be less relevant under the present conditions and the hydroxyl radical was found in relatively high concentrations as discussed above, the peak at m/z 830.5 detected by mass spectrometry that may be formed via reaction with either O_3_ or ^·^OH, we suppose the hydroxyl radical to be the responsible candidate. However, the model used separated the electrode supported POPC bilayer from the short-lived reactive species that are generated or deposited at the gas–liquid interface. Due to its short lifetime, the ^·^OH radical, but also atomic oxygen or singlet oxygen should not be able to cross the bulk of the liquid to attack the POPC molecule. It was suggested, that via bond flipping mechanism the ^·^OH radical might reach into the bulk of liquids^92^, allowing the attack on the lipid. In addition, sub-stoichiometric amounts are assumed sufficient to start a radical chain reaction^93^, leading to the observed oxidation pattern. Furthermore, the distance of 1.8 cm between the gas–liquid interface and the lipid bilayer could be a reason for a different effect on the lipid bilayer (Fig. 3), but a similar concentration of reactive species after 20 and 30 min of treatment (Fig. 9). The 10 min more time could be sufficient for a larger amount of reactive species to reach the lipid bilayer. Still, the long treatment times indicate the limited probability for short-lived species to reach the POPC bilayer. It would be of interest to test the above-mentioned sources that generate atomic oxygen more significantly in the model developed here, as this would allow increasing knowledge on lipid oxidation products and further contribute to the models applicability to study reactive species.

The concentration of hydrogen peroxide (H_2_O_2_), a major long-lived reactive species deposited by noble gas driven CPPs in contact to ambient air^94,95^, increases with treatment time (Fig. 9d), peaking at a concentration of 125 ± 3 µM after 20 min treatment. Similar to BMPO/TEMPD adducts this indicates the presence of consuming reactions leading to the observed saturation of H_2_O_2_ deposition. One of these reactions is the formation of peroxynitrite under lower pH conditions that may occur especially at the gas–liquid interface^96^. In electrochemical experiments (Fig. 3), even 200 µM of hydrogen peroxide showed a negligible effect on the lipid bilayer integrity, returning the focus to the short-lived species as the responsible reaction partners.

## Conclusions

In this work, a combination of electrochemistry and mass spectrometry was developed to study the effect of reactive oxygen species (ROS) generated by CPP on membrane. A supported lipid bilayer formed by the potential assisted vesicle fusion on a gold electrode was established as a model to study the impact of ROS on lipid membrane integrity. The protective role of membrane embedded antioxidant molecules could be elucidated and it is shown to be dependent not only on the well-stablished scavenging of reactive species, but also on structural effects of Q10 on the membrane yielding improved mechanical properties. It can be speculated that other molecules and lipids known to increase the membrane stability may have similar protective roles (e.g. cholesterol, PE-lipids, etc.). Furthermore, this experimental strategy was employed to investigate the chemical potential of cold plasma derived reactive species and their influence on the permeability of lipid bilayers. In contrast to numerous other approaches, the here applied model precluded chemical reactions of the plasma generated species with the target molecules at the gas–liquid interface, but support the observation of subsequent bulk reactions. Candidate reactive species active in the liquid bulk are ^·^OH radicals generated at the interface and active in the bulk via bond flipping mechanisms (^·^OH), and O_2_^·−^/HO_2_^·^ radicals. Atomic oxygen contributes via ^·^OH radical formation. Ozone, another potential candidate to explain the lipid oxidation and subsequent increase in membrane permeability, was ruled out by EPR measurements. Long-lived species such as H_2_O_2_ were ineffective in the model, indicating the role of short-lived species generated by the plasma treatment. The findings are relevant in underpinning the application of cold physical plasmas in cancer treatment, highlighting the difference of membrane stability and antioxidant production for cancer and healthy cells. This study already provide some molecular level insights into the mechanisms of extracellular ROS interacting with the lipid bilayer and membrane during cancer treatment by plasma, bridging towards photodynamic therapy and other (pro-oxidant) neoplastic therapies. In addition, the developed model using a combination of electrochemistry and mass spectrometry, can be used as a general method to study the effect of ROS generated by other therapeutic methods on membrane such as photodynamic therapy and irradiation. Studies including transmembrane proteins to investigate their role in membrane oxidation and permeability will be the subject of our future investigation.

## Experimental

### Materials

Dry powder of 1-palmitoyl-2-oleoyl-sn-glycero-3-phosphocholine (POPC) was from Avanti Polar Lipids (Alabaster, AL). Ubiquinone-10 (Q10), methyl-tert-buthylether (MTBE), chloroform, 2,2,6,6-tetramethyl-4-piperidone hydrochloride (TEMPD–HCl, purity 98.4%), HEPES buffer, and Ammonium formate (MS-grade) were purchased from Sigma-Aldrich. Potassium ferrocyanide (K_4_[Fe(CN)_6_]), disodium hydrogen phosphate (Na_2_HPO_4_), and sodium dihydrogen phosphate (NaH_2_PO_4_) were obtained from Merck. Ammonium acetate was purchased from Roth. Sodium sulfate was purchased from VWR. Formic acid (MS grade) was obtained from Fluka. 5-tert-Butoxycarbonyl-5-methyl-1-pyrroline-N-oxide (BMPO, purity 99.9%) was purchased from Dojindo Laboratoire. Water, methanol, isopropanol (all from ChemSolute) were of MS grade. All chemicals employed were used without further purification.

### Plasma Source and treatment procedure

Reactive oxygen and nitrogen species were generated by a cold physical plasma source, the kINPen (neoplas, Germany). This plasma jet consists of a central pin-type electrode powered by a voltage of 2–6 kV with a frequency of 1 MHz. Argon was used as feed gas (3 standard litres per minute)^97^. For all experiments, a direct treatment regimen was chosen and achieved by exposing 10 mL of water covered solid supported lipid bilayer to the plasma effluent at a distance of 9 mm to the aqueous surface. The movement of plasma jet is performed manually above the water surface (Fig. 1).

### Liposome preparation and characterization

POPC and POPC:Q10 (25:1 mol ratio) liposomes were prepared by sonication. The desired amount of lipid was dissolved in chloroform. For Q10 containing liposomes the necessary amount was added from a stock solution (Q10 stock: 1 mg mL^−1^ in 1:1 chloroform:ethanol). The solvent was evaporated under a constant stream of nitrogen 5.0 (Air Liquide) yielding a homogenous lipid film. Remaining traces of solvent were removed by placing the samples under vacuum overnight. The lipid film was thereafter suspended in deaerated 50 mM phosphate buffer (pH 7.4) and sonicated with a tip-sonicator for 45 min in ice yielding small unilamellar vesicles (SUVs). Afterwards, the suspension was centrifuged for 10 min at 14,000 rpm to remove titanium debris produced during the sonication procedure^66^.

The size distribution of the obtained SUVs was measured by dynamic light scattering (DLS using a NICOMP 380 Particle Sizer (Particle Sizing Systems, Port Richey, FL, US). The final lipid concentration in the samples was 0.5 mg mL^−1^.

The structure and morphology of the liposomes were studied with cryogenic transmission electron microscopy (Cryo-TEM). A Zeiss TEM Libra 120 instrument (Carl Zeiss AG, Germany) was used for analysis, which operates at 80 kV in loss-free bright field mode. A BioVision Pro-SM Slow Scan CCD camera (Proscan elektronische systeme GmbH, Germany) was used to acquire the digital images under low dose conditions. All liposome preparations had a lipid concentration of 1–10 mM. The samples were prepared before microscopy based on a protocol by Almgren and co-workers^98^. Briefly, after addition of a microliter sample, a thin film was prepared on a copper carrier, which was reinforced with a porous polymer film. It is then rapidly vitrified in liquid ethane and transferred to the electron microscope. The sample preparation was carried out at 25 °C and 98–100% relative humidity in a custom-built climate chamber. The samples were protected from atmospheric conditions during the Cryo-TEM studies^32^.

### Preparation and characterization of ultra-flat gold surfaces

The gold surface was cleaned based on a protocol by (Thal et al.)^99^. Briefly, a polycrystalline gold electrodes (Ø 2 mm; Metrohm, Germany) were mechanically polished with Al_2_O_3_ powder (Buehler; grain sizes 300 and 50 nm), and then the rest of alumina particles were removed by sonication from the surface of electrode. Afterwards, electrodes were electrochemically cleaned by cyclic voltammetry from 0 to 1.5 V in 0.1 M H_2_SO_4_ at 0.1 V s^−1^ until a stable voltammogram was obtained (40 ± 10 cycles). Gold oxides were removed from the surface by additional 10 cycles in 0.1 M H_2_SO_4_ from 0.75 to 0.2 V at 0.1 V s^−1^. The roughness of gold surface was calculated based on root mean square (RMS) by atomic force microscopy (AFM) data using gwyddion 2.41 software. All solutions were prepared in high purity Milli-Q water with a resistivity of 18.2 MΩ cm. The electrolytes were deaerated by nitrogen purging before the electrochemical measurements.

### Fabrication of solid supported lipid bilayer

Lipid bilayer on gold surface was formed by slow potential scans (5 mV s^−1^ cycling between 0.4 and − 0.8 V vs Ag/AgCl for at least 4 h) after addition of the freshly prepared liposome suspension to the electrochemical cell**.** After formation of the lipid bilayer on gold electrode, the remaining liposome suspension in the cell was replaced by buffer and water for further experiments^63^.

### Electrochemical measurements

Voltammetric measurements were performed as previously published with a 663 VA stand in combination with Autolab PGSTAT 20 and Eco Chemie IME 303 (Metrohm AG)^63^. The modified gold electrode served as the working electrode, a platinum electrode served as counter electrode and an Ag/AgCl (3 M KCl) electrode as reference. Data were recorded with NOVA 2.0 software (Metrohm AG). The electrolyte solution in all electrochemical experiments was a 10 mM solution of K_4_[Fe(CN)_6_] in 50 mM phosphate buffer (pH 7.4). Cyclic and differential pulse voltammograms were recorded in the potential range from − 0.3 to + 0.7 V vs. Ag/AgCl and 50 mV s^−1^ as a scan rate. Data analysis was performed with OriginPro 2016 (OriginLab Cooperation). Each experiment was repeated at least three times (n = 3).

### Lipid bilayer extraction

POPC and POPC:Q10 lipid bilayer before and after plasma treatments were extracted by MTBE from the surface of gold electrode. Residual water was removed from the combined organic phases by adding water-free sodium sulphate, desiccation with nitrogen gas, and the obtained extract was kept at − 80 °C. Prior to liquid chromatography and mass spectrometry, the dried extract was dissolved in 200 µL of Chloroform: Methanol: Isopropanol (1:2:4, v/v) with 5 mM ammonium acetate as additive.

### High-resolution mass spectrometry

Mass spectrometry data was acquired on a high- resolution mass spectrometer (QExactive Plus, Thermo Scientific). Acquisition was carried out in both positive and negative polarity. For direct infusion acquisition, a data-dependent acquisition Top20 experiment was performed with a total run time of 4 min and a polarity switch in the middle of the experiment. Full MS spectra were acquired at a resolution of 280,000 in the scan range between 50 and 1000 m/z. Corresponding fragment spectra were acquired for all masses with a resolution of 70,000 and a stepped normalized collision energy of 25, 27.5, and 30 eV. Dynamic exclusion time was set to 60 s. For reversed phase LC/MS, a Vanquish UHPLC System with an AccuCore C18 column (2.6 × 150 mm, 1.5 µm, Thermo Scientific) setup was used. Separation was achieved by a binary gradient 50% Methanol (A) and 100% Isopropanol + 10 mM ammonium formate (B), both supplemented with 0.1% formic acid at 50 °C. The flow rate was 150 µL min^−1^, starting from 5% B and linear increasing to 100% B over 10 min followed by washing and equilibration step. Injection volume was 1 µL. Every sample was injected twice for both polarities. Samples were measured using a data-dependent Top15 method with a resolution of 70,000 for full scan spectra and 17,500 for fragment scans. Dynamic exclusion time was set to 30 s and scan range set from 100 to 1000 m/z. Direct infusion setup data was analyzed using LipidXplorer Software and Freestyle Software (Version 1.5, Thermo Fisher Scientific)^100^. Data from RP-LC MS measurements was analyzed using LipidSearch (Version 4.1.16, Thermo Scientific).The MarvinSketch© software (version 18.8.0) was used to draw the chemical structure of POPC and its oxidation products.

### Electron paramagnetic resonance

Electron paramagnetic resonance spectroscopy measurements were performed using an X-band (9.87 GHz) EPR spectroscope (EMXmicro, Bruker Biospin GmbH) with the resonator ER 4119HS (Bruker BioSpin GmbH). The following instrument parameters were applied for all measurements: modulation frequency of 100 kHz, modulation amplitude of 0.1 mT, microwave power of 5.024 mW, receiver gain of 30 dB, and time constant of 0.01 ms. In the presented work, the spin trap BMPO (50 mM) for ^·^OH and O_2_^·−^ radicals, and the spin probe TEMPD-HCl (100 mM) for ^·^O, O_2_ (a^1^Δ_g_), or O_3_ were used. Their concentrations were chosen so that the trapping agent was offered in excess. To allow sample handling and ensure comparability, the delay between CPP treatment and EPR measurement was fixed to three minutes. Measurements were performed as triplicates for each sample. Prior to a measurement series, the untreated solution were used as background control. The spectrum of the untreated control was subtracted from the measured EPR signal. The evaluation of the EPR spectra was performed by assistance of the evaluation software (Xenon software with Xenon Spin Counting module, Bruker BioSpin GmbH). The spectroscope was calibrated and absolute spin numbers could be gained. The calibration was performed by placing an alanine spin counting calibration sample into the EPR, measuring its spectrum and calculating the absolute number of spins in the sample. This number was compared with the suppliers number (Bruker Biospin GmbH) and a correction factor was determined. More details about the EPR procedure and the calibration can be found in Tresp et al.^101^.

### Colorimetric hydrogen peroxide assay

Hydrogen peroxide (H_2_O_2_) was measured with a Pierce Quantitative Peroxide Assay Kit (Thermo Scientific) based on the manufacturer’s protocol. The simplified reaction sequence involves the oxidation of ferrous (Fe^2+^) to ferric (Fe^3+^) ions by hydrogen peroxide with the subsequent binding of the Fe^3+^ ion to the ferric-sensitive dye xylenol orange, yielding an orange to purple complex which can be detected photometrically (Tecan Infinite M200 Pro, Tecan) at 595 nm^102^. Each 96-well plate contained a standard curve (0–150 μM, in triplicates) and the samples (n = 4, each sample in triplicates).

## Supplementary information

Supplementary Information
