# Author Correction: A combination of electrochemistry and mass spectrometry to monitor the interaction of reactive species with supported lipid bilayers

**DOI:** 10.1038/s41598-021-91696-0

**Published:** 2021-06-11

**Authors:** M. Ravandeh, H. Kahlert, H. Jablonowski, J.-W. Lackmann, J. Striesow, V. Agmo Hernández, K. Wende

**Affiliations:** 1 Institute of Biochemistry, University of Greifswald, Felix-Hausdorff-Str. 4, 17489 Greifswald, Germany; 2 Leibniz-Institute for Plasma Science and Technology, ZIK Plasmatis, Felix-Hausdorff-Str. 2, 17489 Greifswald, Germany; 3 Department of Chemistry-BMC, Uppsala University, Husargatan 3, 75123 Uppsala, Sweden; 4 Department of Pharmacy, Uppsala University, Husargatan 3, 75123 Uppsala, Sweden

Correction to: *Scientific Reports* 10.1038/s41598-020-75514-7, published online 29 October 2020

The Article contains errors in the Reference list. The authors omitted the below papers, which are listed as References 103–104. These should be cited in the Introduction section as below:

“Among these, the application of electrochemistry is one of the best, because the interaction between reactive species and biomolecules based on electron transfer reactions can be easily monitored^64,65^.”

should read:

“Among these, the application of electrochemistry is one of the best, because the interaction between reactive species and biomolecules based on electron transfer reactions can be easily monitored^64,65,103,104^.”
