# Supplementary material for: A combination of electrochemistry and mass spectrometry to monitor the interaction of reactive species with supported lipid bilayers
Source: Sci Rep. 2020 Oct 29;10:18683. doi: 10.1038/s41598-020-75514-7 (PMC7596530; doi:10.1038/s41598-020-75514-7)
Supplement: Supplementary file 1 — Supplementary Information [file 41598_2020_75514_MOESM1_ESM.pdf]

A combination of electrochemistry and mass spectrometry to monitor the interaction of reactive species with supported lipid bilayers

M. Ravandeh, H. Kahlert, H. Jablonowski, J.-W. Lackmann, J. Striesow, V. A. Hernández, K. Wende

Supplementary information

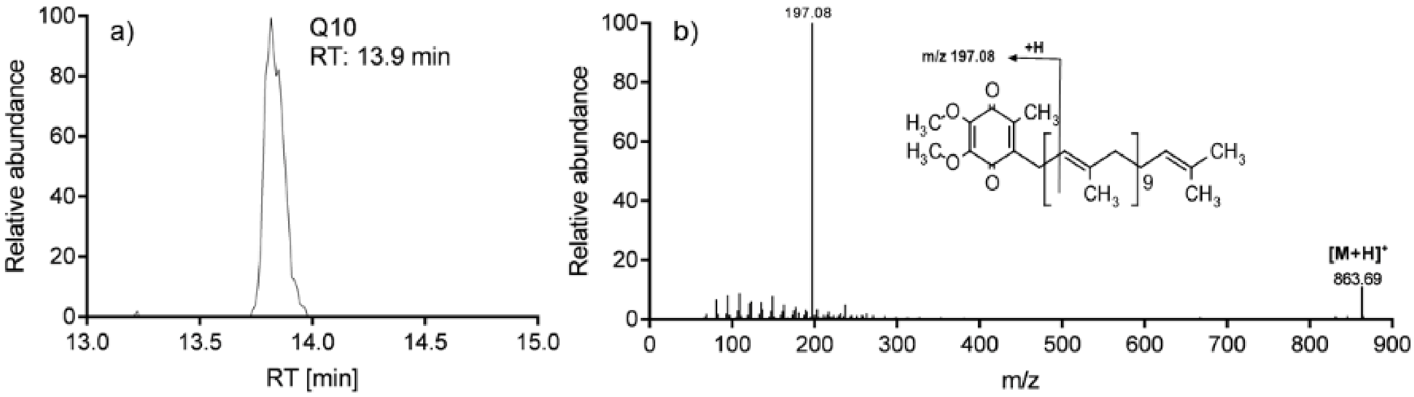

Figure S1: Extracted ion chromatogram of Q10 measured in positive mode. Detected as protonated precursor ion at 863.69 m/z (a), MS1/MS2 spectrum of protonated Q10 showing a diagnostic fragment ion at m/z 197 (b)

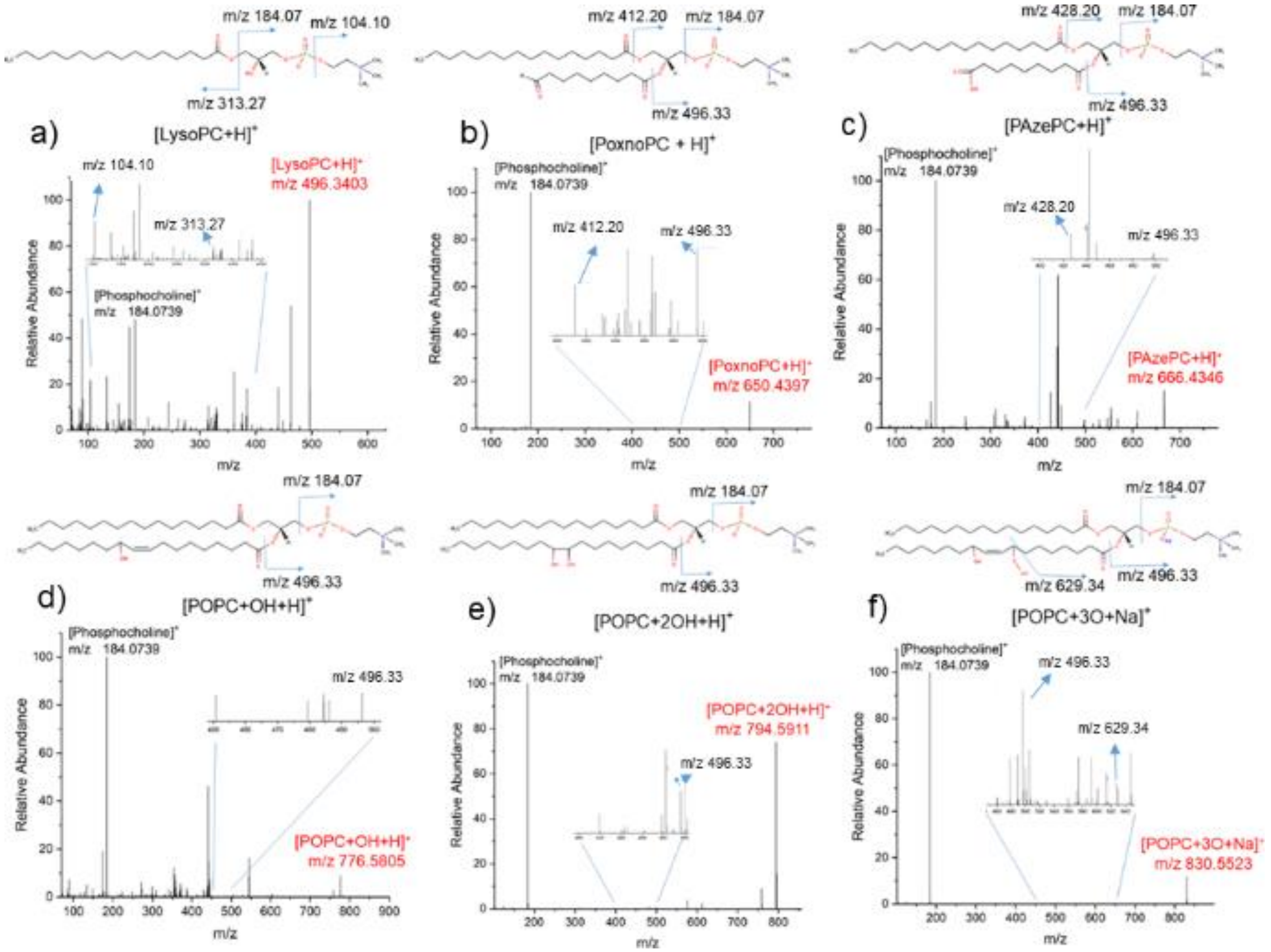

Figure S2: Tandem mass spectrometry of POPC oxidation products in positive mode: a) $[LysoPC + H]^+$ (m/z 496.3403) ($C_{24}H_{51}NO_7P$), b) $[PoxnoPC + H]^+$ (m/z 650.4397) ($C_{33}H_{65}NO_9P$), c) $[PAzePC + H]^+$ (m/z 666.4346) ($C_{33}H_{65}NO_{10}P$), d) $[POPC+OH + H]^+$ (m/z 776.5805) ($C_{42}H_{83}NO_9P$), e) $[POPC+2OH + H]^+$ (m/z 794.5911) ($C_{42}H_{85}NO_{10}P$), and f) $[POPC +3O + Na]^+$ (m/z 830.5523) ($C_{42}H_{82}NNaO_{11}P$)

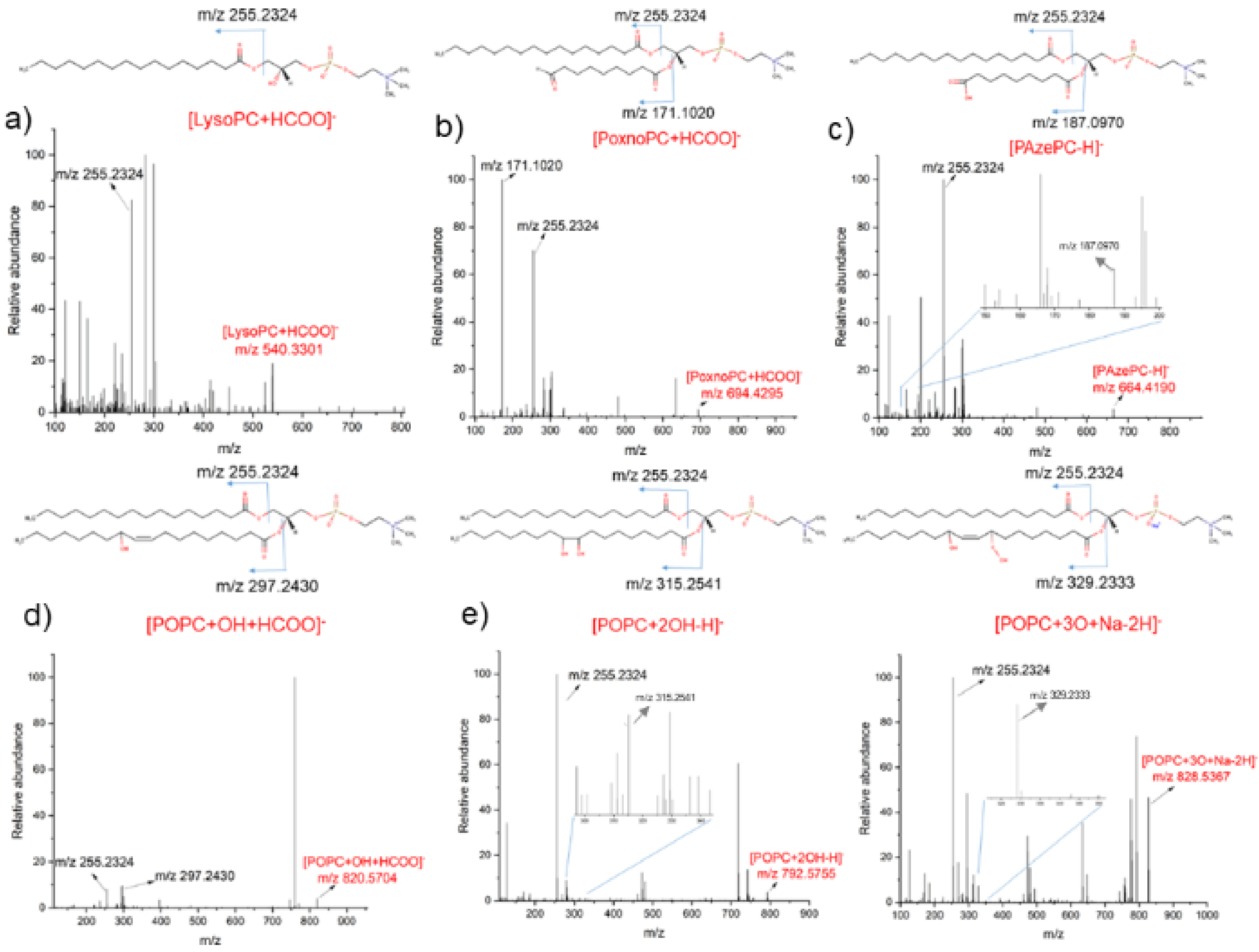

Figure S3: Tandem mass spectrometry of POPC oxidation products in negative mode: [LysoPC + HCOO]$^-$ (m/z 540.3301) ($C_{25}H_{51}NO_9P$), b) [PoxnoPC + HCOO]$^-$ (m/z 694.4295) ($C_{34}H_{65}NO_{11}P$), c) [PAzePC - H]$^-$ (m/z 664.4190) ($C_{33}H_{63}NO_{10}P$), d) [POPC+OH + HCOO]$^-$ (m/z 820.5704) ($C_{43}H_{83}NO_{11}P$), e) [POPC+2OH - H]$^-$ (m/z 792.5755) ($C_{42}H_{83}NO_{10}P$), and f) [POPC +3O + Na – 2H]$^-$ (m/z 828.5367) ($C_{42}H_{80}NNaO_{11}P$)
